# Topography and Anatomical Variations of the Axillary Artery

**DOI:** 10.1155/2021/6393780

**Published:** 2021-05-24

**Authors:** Kiwook Yang, Hyunsu Lee, In-Jang Choi, Woonhyeok Jeong, Hong-Tae Kim, Qun Wei, Jae-Ho Lee

**Affiliations:** ^1^Department of Anatomy, School of Medicine, Keimyung University, Daegu, Republic of Korea; ^2^Department of Plastic and Reconstructive Surgery, School of Medicine, Keimyung University, Daegu, Republic of Korea; ^3^Department of Anatomy, School of Medicine, Catholic University of Daegu, Daegu, Republic of Korea; ^4^Department of Biomedical Engineering, School of Medicine, Keimyung University, Daegu, Republic of Korea

## Abstract

Knowledge of anatomical variations of the limb's main arteries is significant for the clinicians. Thus, this study is aimed at examining the branching pattern and anatomical variations of the axillary artery. We conducted research on 59 upper limbs of adult human donated cadavers. All axillary artery branches' origins were assessed, and the correlations between points of origins and variations of specific branches were evaluated. The average length of the axillary artery was found to be 11.22 cm, and this length was defined as reference line. Based on this reference line, the first, second, and third parts were 37.56%, 39%, and 30.05%, respectively. The STA was originated from 25.11%. The TAA and LTA were 42.67% and 54.82%, respectively. The SSA, ACHA, and PCHA were 64.72%, 83.89%, and 84.53%, respectively. The origin of LTA was correlated with that of SSA (*R* = 0.473, *P* < 0.05) and AHCA (*R* = 0.307, *P* < 0.05), respectively. And there was a positive correlation between AHCA and PHCA (*R* = 0.705, *P* < 0.05). The number of branches ranged from 3~6, and 9 types were shown. The most frequent branching pattern was common origin of the LTA and SSA (22/59). And AHCA and PHCA were originated together in 19 cases, and both patterns were combined in 12 cases. TTA and LTA branched together in 9 cases, and common trunk for the SSA, PHCA, and AHCA was found in 2 cases. According to this pattern, the origin of LTA and PCHA was significantly different. This information is particularly useful for surgeons and clinicians.

## 1. Introduction

In humans, the axillary artery is the direct continuation of the subclavian artery. It provides the blood supply to the lateral wall of the chest, axilla, and upper extremity. It originates from the side edge of the first rib before it is called the subclavian artery [1]. The axillary artery often consists of three parts depending on the artery location relative to the pectoralis minor muscle, which is superficial to the artery. The first part is proximal, the second part is posterior, and the third part is distal to this muscle. In normal anatomy, six branches arise from the axillary artery. The first branch, the superior thoracic artery (STA), originates from the first part. The second and third branches, the thoracoacromial artery (TAA) and lateral thoracic artery (LTA), originate from the second part. The fourth, fifth, and sixth branches, the subscapular artery (SSA), anterior circumflex humeral artery (ACHA), and posterior circumflex humeral artery (PCHA), respectively, originate from the third part [2]. The origin and morphology of the axillary artery branches are diverse, and numerous studies report various variations. In most of these studies, the axillary artery branches were often found to be branched together [3–6].

Details regarding deviation from the normal arterial pattern and variations of the axillary artery are crucial for anatomists, plastic and orthopedic surgeons, vascular radiologists, and interventional cardiologists [7–11]. In addition, injuries of the brachial plexus are quite common and require exploration and repair. The axillary artery variation is a concern during these procedures [12–14]. For better clinical procedures, detailed knowledge of anatomical relationships of the axillary artery and distribution of its branches is crucial [15–18]. Therefore, topography of the axillary artery and its branches should be studied and their association needed to be discussed.

In this study, the branching patterns and origin of the axillary artery and its correlation were analyzed in 59 upper limbs of Korean cadavers. This information has practical implications and can be helpful for accurate diagnostic interpretation. This study is aimed at determining the branching patterns and variations of the axillary artery.

## 2. Materials and Methods

### 2.1. Prevalence and Length of the Axillary Artery

In this study, 59 upper limbs (from 30 donated cadavers, 30 right and 29 left) were dissected. Each cadaver was placed in a supine position with arms abducted and palms facing up. The skin, superficial fascia, and adipose tissue were removed to expose the axillary artery. The pectoralis major and pectoralis minor muscles were dissected. After the brachial plexus, axillary vein, teres major muscle, pectoralis major muscle, and pectoralis minor muscle were dissected from each muscle and fascia, the axillary artery was identified. After identification, the length between the lateral border of the first rib and the inferior border of the teres major muscle was measured by digital calipers (NA500-300S, Blue Bird, Korea) and defined as the reference line [1, 2]. The branching patterns and variations of the axillary artery were analyzed. The length from the lateral border of the first rib to the branching points of the axillary artery was compared with the length of the reference line (in percentile).

### 2.2. Topography of the Axillary Artery

The branching points of the axillary artery were analyzed and divided into three parts. Each branching point was defined as the length from the lateral border of the first rib to the branching point. All branching points were calculated with respect to the reference line in percentile. Their branching patterns were classified according to the branching combination. The difference in the branching point of each artery was compared according to the branching pattern, sex, and left and right upper limbs. And the correlation between the branching points and three parts of the axillary artery was analyzed to sex and left and right upper limbs.

### 2.3. Statistical Analysis

All statistical analyses were conducted using SPSS (version 20.0, IBM SPSS®; Chicago, IL). The Pearson correlation test and Kruskal-Wallis test were used to analyze the relationship between the variations of the axillary artery. *P* values < 0.05 were considered to indicate statistical significance.

## 3. Results

### 3.1. The Origin and Length of the Axillary Artery

**Figure 1:**
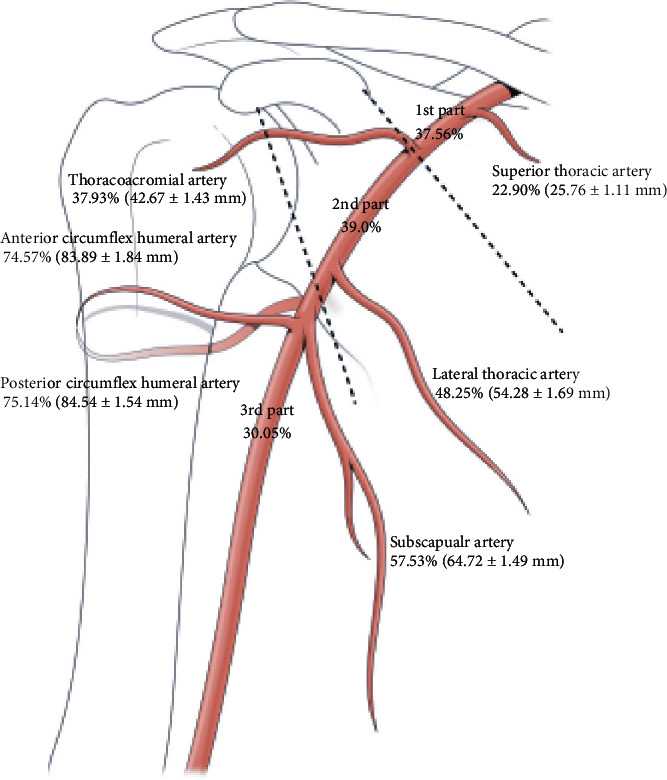
Topography of the axillary artery.

The average length of the axillary artery was found to be 112.50 mm (reference line; median: 110.57 mm; range: 80–150 mm). With respect to reference line, the length of the starting point of the second part of the axillary artery was 33.39% (median: 36.55%; mean length: 37.56 ± 1.28 mm). The length of the starting point of the third part of the axillary artery was 69.51% (median: 71.94%; 78.20 ± 1.78 mm). STA originated from 22.90% (median: 24.30%; 25.76 ± 1.11 mm). TAA and LTA originated from 37.93% (median: 43.63%; 42.67 ± 1.43 mm) and 48.25% (median: 55.11%; 54.28 ± 1.69 mm), respectively. SSA, ACHA, and PCHA originated from 57.53% (median: 63.96%; 64.72 ± 1.49 mm), 74.57% (median: 86.14%; 83.89 ± 1.84 mm), and 75.14% (median: 86.52%; 84.54 ± 1.54 mm), respectively (Figure 1).

### 3.2. Correlation among Branches of the Axillary Artery

**Figure 2:**
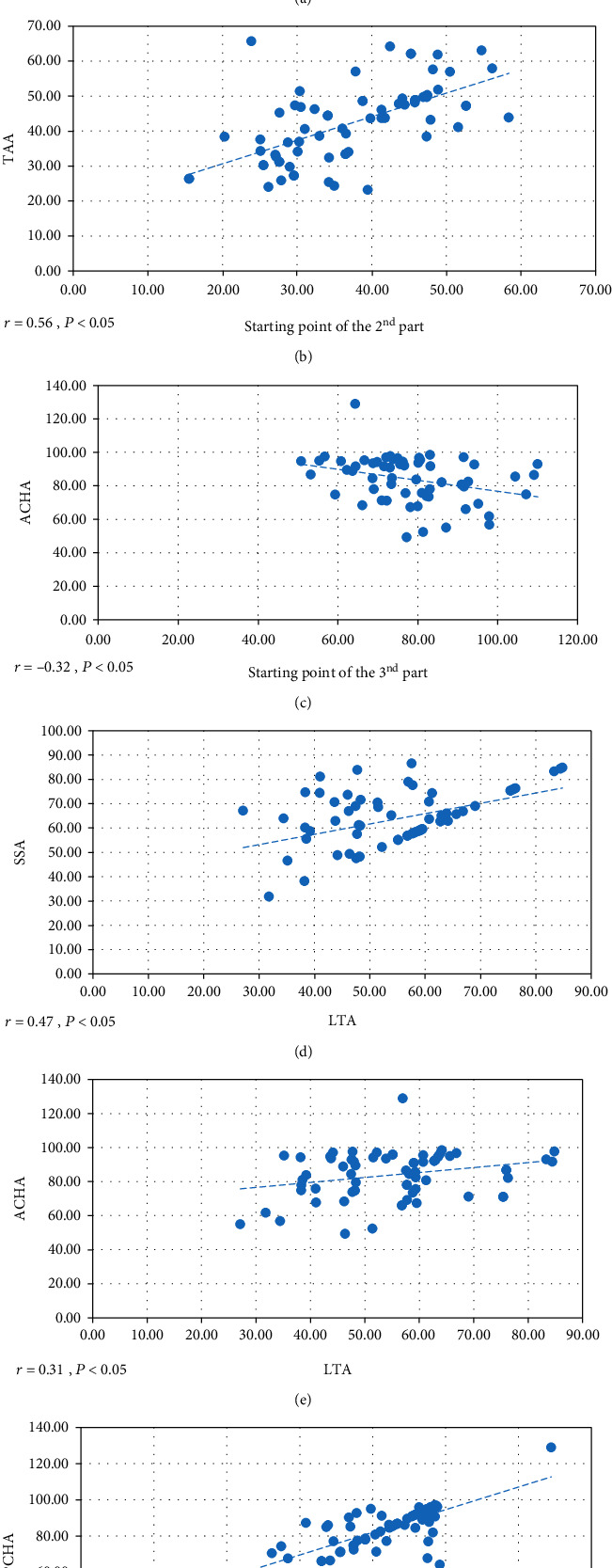
Correlation result. (a) Starting point of the 2^nd^ part and the origin of superior thoracic artery (STA). (b) Starting point of the 2^nd^ part and the origin of thoracoacromial artery (TTA). (c) Starting point of the 3^rd^ part and the origin of anterior circumflex humeral artery (ACHA). (d) The origin of lateral thoracic artery (LTA) and the origin of subscapular artery (SSA). (e) The origin of lateral thoracic artery (LTA) and the origin of ACHA. (f) The origin of ACHA and the origin of posterior circumflex humeral artery (PCHA).

The correlation between origin points of branches and parts was analyzed. The length of the starting point of the second part of the axillary artery positively correlated with the origin of STA (*r* = 0.26, *P* < 0.05, Figure 2(a)) and TAA (*r* = 0.56, *P* < 0.05, Figure 2(b)). The length of the starting point of the third part of the axillary artery negatively correlated with ACHA (*r* = −0.32, *P* < 0.05, Figure 2(c)). LTA distribution positively correlated with SSA (*r* = 0.47, *P* < 0.05, Figure 2(d)) and AHCA (*r* = 0.31, *P* < 0.05, Figure 2(e)). There was a positive correlation between AHCA and PHCA (*r* = 0.71, *P* < 0.05, Figure 2(f)). Other branches did not have any correlation among themselves.

### 3.3. Branching Pattern of the Axillary Artery

**Figure 3:**
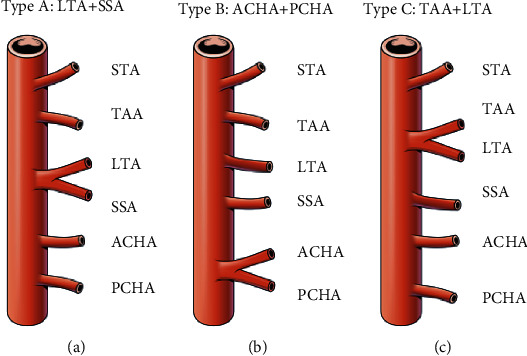
Frequent branching pattern of the axillary artery. (a) Type A: common origin for lateral thoracic artery (LTA) and subscapular artery (SSA). (b) Type B: common origin for anterior circumflex humeral artery (ACHA) and posterior circumflex humeral artery (PCHA). (c) Type C: common origin for thoracoacomial artery (TAA) and LTA.

We examined the branching patterns of the axillary artery to examine the relationship between the origin of the branching point and the distribution pattern. Considering every branch given off directly by the axillary artery, the branches were 3–6 in number. Typical variant of the axillary artery was found in 15 out of 59 cases (25.4%). The most frequent variant of branching pattern of the axillary artery involved 22 cases (37.3%) in which LTA and SSA branched together (Figure 3(a)), followed by a common branch for AHCA and PHCA in 19 cases (Figure 3(b)). One trunk for LTA and SSA and one trunk for ACHA and PCHA were found together in 12 cases. TAA and LTA were branched together in 9 cases (Figure 3(c)). SSA, PHCA, and AHCA originated from a common trunk in 2 cases. TAA, LTA, and PHCA were branched together in one case. LTA, SSA, and PHCA were branched together in one case. TAA, LTA, and SSA were branched together in one case. SSA and PHCA were branched together in one case. There was no statistically significant difference with respect to sex and left and right upper limbs.

### 3.4. Topographical Changes in Branches according to the Branching Pattern of the Axillary Artery

Excluding rare patterns, the branching pattern was classified into 5 types: typical, common branch of LTA and SSA (type A), common branch of ACHA and PCHA (type B), common branch of TAA and LTA (type C), and two common trunks for LTA/SSA and ACHA/PCHA (combined types A and B). The origin of 6 branches was analyzed according to these branching patterns (Table 1). Compared to typical pattern, STA originated distally in atypical types; however, the difference was not significant (*P* = 0.330). LTA was originated more proximally compared to other types (*P* = 0.004). And the origin of PCHA was more distal in atypical pattern, especially type B (*P* = 0.021). Other variable did not have any correlation.

## 4. Discussion

In this study, the length of the axillary artery as reference line was found to be 80–90 mm in 4, 90–100 mm in 15, 100–110 mm in 9, 110–120 mm in 15, and >120 mm in 16 cases. With respect to this reference line, the axillary artery was divided into three parts. The first, second, and third parts were 33.39%, 36.12%, and 30.49%, respectively. The average length of the origin of the six branches was also investigated. Interestingly, SSA originated from 57.53%, and it included in the second part of the axillary artery. Most textbooks describe that SSA originates from the third part; however, we showed different results.

The anatomical variation of the axillary artery is clinically important because various blood vessels and nerves pass through it [15–17]. In particular, branches of the axillary artery are quite diverse from the bases, with two or three arteries branching together. These branches correlate with each other based on their origin. As expected, there was a positive correlation between AHCA and PHCA. The origin of LTA was positively correlated with that of SSA. Interestingly, the origin of PCHA was negatively associated with the length of the third part. The origin of the branches of the axillary artery varied according to the branching patterns. These data indicated hypothesis that these arteries develop simultaneously, and their branching patterns may not be random.

Nine types of branching patterns of the axillary artery were found. This result was comparable with those of previous studies [5, 19, 20]. The patterns and origins of the axillary artery branches have been investigated, but studies about the correlation among these patterns and origins were lacking in other studies [3, 5, 6, 20–22]. Remarkably, the origins of LTA and PCHA were significantly different according to their branching patterns, suggesting that the morphology of the axillary artery during development influences the branching point. When LTA originated with TAA, its origin was more proximal, and when PCHA originated with ACHA, its origin was more distal. Until now, the arterial division was known as random. However, our results proposed hypothesis that the branching pattern and branching point are strongly associated and have unidentified regulation that one artery can correlate the position of another artery.

Unusual variants of the axillary artery branching or formation may also occasionally exist. For instance, the axillary artery may give rise to the radial artery of high origin (known as brachioradial artery) or to the superficial ulnar artery [23–25]. In our study, we did not find those rare variants. However, knowledge of all unexpected deviations from the typical axillary artery branching pattern is fundamental from the clinical perspective. All variations have an embryological background since upper limb vasculature develops from the primitive capillary plexus, which gives the potential to various blood flow pathways formation [20, 25].

Studies about the patterns and origins of the axillary artery branches and their correlation are important for clinicians [4–6]. Patients with severe trauma to the shoulder and upper chest should be clinically assessed for vascular damage and musculoskeletal evaluation [12–17]. In addition, it will be helpful to recognize and understand such a variation, even in radiographic procedures, to increase the accuracy of the technique and to reduce unnecessary complications [26, 27]. Based on an accurate understanding of the axillary artery branching, as in this study, understanding the branching patterns and local anatomical variations may be helpful in the accurate assessment and proper management of the injured area [28].

This study investigated the patterns and variations of the axillary artery to provide useful information to clinicians especially dealing with the axillary region in the case of reconstructive surgery. Our research involved the axillary artery exclusively. We did not perform a whole-body study of anatomical variations. Kahn et al. [29] suggest that a whole-body study of arterial variants in a single anatomical donor should be performed to assess the coincidence of vascular variations. Relations with the brachial plexus and its branches also were not assessed. However, in the presented report, we provided a detailed description of the variations of the axillary artery branching pattern. As the authors, we hope that repeated observations on anatomical variations deepen existing knowledge, help overcome the subjective aspect in the description made by individual researchers, and can be useful for clinicians in their daily practice.

## Figures and Tables

**Table 1:** The origin of the branches of the axillary artery according to the branching pattern.

	Typical (percentile)	Atypical type (percentile)			
		Type A	Type B	Type C	Types A and B
STA	22.6 ± 6.7	23.6 ± 10.5	29.6 ± 7.7	27.9 ± 11.0	27.4 ± 7.7
TAA	44.6 ± 7.7	43.0 ± 14.0	39.3 ± 12.9	45.6 ± 9.3	41.7 ± 10.9
LTA^∗∗^	55.7 ± 13.1	54.7 ± 17.8	50.5 ± 6.4	48.7 ± 9.5	58.6 ± 10.4
SSA	65.9 ± 10.9	59.3 ± 15.8	65.7 ± 9.7	69.7 ± 10.3	61.4 ± 9.6
ACHA	79.6 ± 14.4	81.2 ± 15.3	98.2 ± 13.8	84.39 ± 7.8	85.3 ± 8.5
PCHA^∗^	80.0 ± 12.8	88.5 ± 6.9	97.8 ± 13.9	84.3 ± 7.1	85.4 ± 8.4

^∗^ *P* < 0.05, ^∗∗^*P* < 0.01.

## Data Availability

All the data in my manuscript is available.
